# The goldmine of patient satisfaction-interaction and communication in the context of infectious diseases

**DOI:** 10.25122/jml-2021-0258

**Published:** 2021

**Authors:** Norbert Dacian Stenczel, Traian Soare, Ciprian Ianovici, Silvia Sovaila, Iuliana Raluca Gheorghe, Victor Lorin Purcarea

**Affiliations:** 1. Department of Healthcare Marketing and Medical Technology, Carol Davila University of Medicine and Pharmacy, Bucharest, Romania; 2. Infectious Diseases and Psychiatry Hospital, Baia Mare, Romania

**Keywords:** infectious diseases, patient satisfaction, health care services

## Abstract

This study aimed to investigate the patient satisfaction level in terms of communication and interaction with the physicians from a Romanian Infectious Disease hospital. The objectives of the study were related to the identification of the general level of satisfaction of the patients, the evaluation of the physicians’ interaction type with their physicians, by using specific behavioral variables, such as respect and attention, as well as to determine the physician-patient communication quality. The data were collected with a self-administered questionnaire and were analyzed using IBM SPSS version 25. The sample comprised 82 patients who were hospitalized in the Department of Infectious Diseases. The quantitative variables were evaluated with the Shapiro-Wilk test and were described by the means and standard deviations, while the qualitative data were described by using the absolute values and percentages. The vast majority of patients were aged between 18–28 years old, were mostly females from urban areas, and 41.5% had university degrees. The outcomes of the study revealed that the general satisfaction of the patients, from a communication perspective, was reflected in the perceived and provided quality of information about the treatment. Although the vast majority of patients highly appreciated the interaction and the communication with their physicians, some individuals felt the need to read more information about their disease, especially from online sources, and they would have preferred their physicians to recommend trustful websites or health care platforms.

## Introduction

Patient satisfaction is a complex concept that is frequently used in clinical practice. More precisely, satisfaction is the unit of similarity between patient expectations and their received genuine health care services [1]. In fact, satisfaction provides insight regarding various health care characteristics, including the effectiveness of the provided care and the patient’s level of understanding. Despite the entropy of information in the physician-patient relationship [2], from a patient’s perspective, satisfaction may be measured in terms of communication, interaction (politeness and respect), and the functional features of the health care service. Evaluating the extent to which patients are satisfied with the health care services is clinically significant because satisfied patients show a higher level of adherence to the prescribed treatment.

An infectious disease is defined as any disease triggered by pathogenic microorganisms, namely, bacteria, fungi, parasites, or even viruses. An individual may get infected with a pathogenic microorganism, either directly or indirectly, passing it in the community or group of individuals [3].

Research showed that infectious disease specialists have different tasks and carry a massive burden as millions of individuals are impacted each year, either by falling ill or dying from an infectious disease. Treating and diagnosing an infectious disease is even more difficult because the symptoms vary greatly and frequently include fatigue and fever. Depending on the gravity of the illness, the global health coverage has faced critical challenges in the pandemic context and, unfortunately, it turned out to be unsuccessful in managing the chronic disease patients with pre-existing conditions such as heart diseases, high blood pressure, diabetes, and kidney disease [4]. During the COVID-19 pandemic, in developed countries, patient needs were managed by telemedicine, which nowadays became vital to delivering infection control methods and compliance to social distancing [5].

This study aimed to investigate the level of patient satisfaction related to the communication and the interaction with the physicians of the Infectious Disease hospital from Baia Mare, Romania. More precisely, the objectives of the case study were:

• Identifying the socio-demographic profiles of patients who were hospitalized in the Department of Infectious Diseases;
• Assessing the patients’ motivations that stood behind the hospitalization in the Department of Infectious Disease, and the number of actions they conducted before it;
• Evaluating the patients’ type of interaction with their physicians by using specific variables, such as respect, patience, attention;
• Determining the physician-patient communication quality at two different time moments;
• Identifying the general level of satisfaction of the patients with the medical employees from a communication perspective.

## Material and Methods

The case study was observational and cross-sectional, and the sampling method was non-probabilistic. The sample of respondents was made up of 82 patients hospitalized in the Department of Infectious Diseases, regardless of gender, age, and level of education.

The data were collected with a self-administered questionnaire consisting of 11 questions, which eased the collection of socio-demographic information. Three questions focused on the aim and the type of hospitalization, and four questions concentrated on the patient interaction with their physicians. Seven questions were about the physician-patient communication and one question related to the satisfaction level of the patients regarding the quality of information, the diagnosis, and the treatment provided by the infectious diseases physicians.

The confidentiality and anonymity of the answers were guaranteed during the data collection phase, with participants acknowledging the scope of the study. Further, the questionnaires were validated by following a qualitative criterion, namely answering all the questions.

The data collection and analysis were conducted using IBM SPSS Statistics version 25. The quantitative variables were evaluated with the Shapiro-Wilk test for checking the distribution type and were described by means and standard deviations, while the qualitative data were described by using the absolute values or percentages.

The independent quantitative variables with nonparametric distribution were validated by using both the Mann-Whitney U test and Kruskal-Wallis H test, followed by the Spearman rho correlation coefficient that aimed to check the correlation between two variables. The independent quantitative variables with parametric distribution were tested using the t-test (Student) or One-way ANOVA. The qualitative variables were tested by using the Fisher Exact test, and the Z tests with the Bonferroni correction were performed to have a more in-depth overview of the qualitative variables. At the same time, the Dunn-Bonferroni post-hoc tests were performed with the scope of having an in-depth overview of the outcomes as well.

## Results

### The results of objective 1

**Figure 1.**
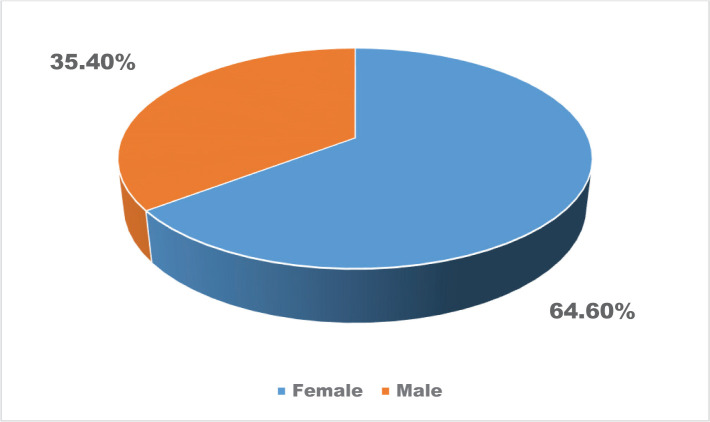
The distribution of the patients’ gender.

**Figure 2.**
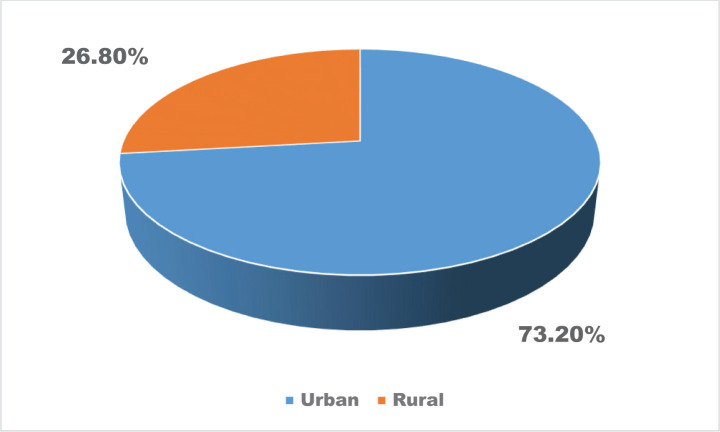
The distribution of the patients’ residential area.

The vast majority of patients who were hospitalized in the Department of Infectious Diseases were aged between 18 and 28 years (35.5%), mainly females (64.6%), from urban areas (73.2%), and 41.5% had university degrees (Tables 1 and 2, Figures 1 and 2).

**Table 1.** The distribution of the patients’ age.

**Age category**	**Number**	**Percentage**
**18–28 years**	29	35.4
**29–39 years**	16	19.5
**40–49 years**	18	22
**50–60 years**	9	11
**Over 60 years**	10	12.2

**Table 2.** The distribution of the patients’ education level.

**Education level**	**Number**	**Percentage**
**Primary school**	14	17.1
**High-school**	21	25.6
**University**	34	41.5
**Post-university**	13	15.9

### The results of objective 2

The vast majority of patients mentioned that the hospitalization motivation was a general bad health status (46.3%) and, at the same time, were admitted at the same hospital (58.5%) but were cared for by a different physician (63.4%). Moreover, the distribution of the patients’ actions before being hospitalized revealed that 18.3% came directly to the hospital, 50% looked for information on the internet about a physician, and 57.3% only searched for information on the internet for similar symptoms (Table 3).

**Table 3.** The distribution of the patients’ actions before being hospitalized.

**Before being hospitalized**	**Number of actions**	**Percentage**
**You have searched on the internet for similar symptoms**	47	57.3
**You have searched on the internet for a treatment that may be administered at home**	40	48.8
**You have searched on the internet for information regarding the physician you came across in the hospital**	41	50
**You have contacted your family physician**	27	32.9
**You came directly to the hospital**	15	18.3

Regarding the identification of the mean number of patients’ actions before hospitalization, a new quantitative variable has been built, which practically reflects the sum of all previous actions of patients before hospitalization. According to the Shapiro-Wilk test (p<0.001), having a nonparametric distribution, for results reporting, the median and the interpercentiles’ interval have been used. Because the median of the number of actions is 3, with variations from 1 to 3 actions, it may be concluded that 75% of the patients who did not come directly to the hospital have conducted between 1 and 3 actions before hospitalization, for instance, searching the internet for symptoms, treatment, a physician or just followed the advice of the family physician (Table 4).

**Table 4.** The number of actions before hospitalization.

**Parameter**	**Mean (SD)**	**Median**	**Minimum**	**Maximum**
**No. of actions (p<0.001)**	2.31 (1.033)	3 (1-3)	1	4

The sum of actions before hospitalization of the patients on age categories is described in Tables 5 and 6. As such, the distribution of the vast majority of groups being nonparametric according to the Shapiro-Wilk test (p<0.01), for the reporting of the results, the mean rank of the parameter should be used (Table 5). According to the Kruskal-Wallis H test, the differences between groups have been statistically significant (p<0.001), and the post-hoc tests showed that the patients with age between 18–28 years (median – 3 actions, mean rank=43.38, p<0.001) or 29–39 years (median – 3 actions, mean rank=36.67, p=0.023) conducted more actions before hospitalization in comparison with the patients older than 60 years (median – 1 action, mean rank=11).

**Table 5.** The sum of actions before hospitalization of the patients who did not come directly to the hospital on age categories.

**Age categories**	**Mean (SD)**	**Median**	**Mean Rank**	P *
**18–28 years (p=0.001)**	2.85 (0.83)	3 (2–3)	43.38	<0.001
**29–39 years (p=0.009)**	2.47 (0.91)	3 (2–3)	36.67
**40–49 years (p=0.005)**	2 (1.03)	2 (1–3)	28.41
**50–60 years (p=0.001)**	1.67 (1.15)	1	22.83

* – Spearman’s rho Correlation Coefficient; ** – Shapiro-Wilk Test.

**Table 6.** The post-hoc tests of the sum of actions before hospitalization of patients who did not come directly to the hospital on age categories.

**Age category**	**18–28 years**	**29–39 years**	**40–49 years**	**50–60 years**	**>60 years**
**18–28 years**	-	1.000	0.104	0.669	<0.001
**29–39 years**	1.000	-	1.000	1.000	0.023
**40–49 years**	0.104	1.000	-	1.000	0.367
**50–60 years**	0.669	1.000	1.000	-	1.000
**>60 years**	<0.001	0.023	0.367	1.000	-

Dunn-Bonferroni Post-Hoc Test

### The results of objective 3

The results of the interaction between physicians and patients have been reflected in respectful behavior and greater attention. Therefore, 96.3% of patients mentioned that their physicians had a respectful behavior towards them, and 92.7% of patients totally agreed with the statement centered on the physicians’ patience in clearly explaining the diagnosis and the treatment to their patients. Also, the vast majority of patients (90.2%) totally agreed with the statement that their physicians had used terms that they easily understood, but some of the patients (1.2%) became careless.

Regarding the degree of care and interest showed by the physicians, most patients confirmed that they were the right ones (96.3%).

A new quantitative variable that reflected the level of satisfaction of the patients regarding their interaction with their physicians has been built. Thus, the new variable, having a nonparametric distribution according to the Shapiro-Wilk test (p<0.001), the reporting of the result has been performed with the help of the median and the interpercentiles’ interval. Table 7 reveals that the vast majority of patients had a maximum level of satisfaction, namely 20 points, and suggested that most patients considered that they had an excellent interaction with their physicians.

**Table 7.** The satisfaction level of the interaction between the patients and their physicians.

**Parameter**	**Mean (SD)**	**Median**	**Minimum**	**Maximum**
**Interaction score**	19,74 (0,62)	20 (20–20)	17	20

### Results of objective 4

**Figure 3.**
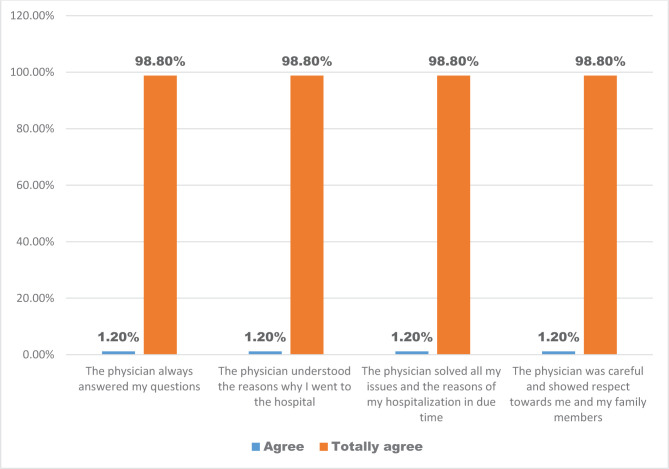
The distribution of the patients’ opinions regarding the quality of the communication between the physicians and the patients.

The quality of the communication between the physician and the patient has been investigated from two different time perspectives: present and future. Hence, most patients agreed that they had a suitable communication with their physicians; for instance, physicians have always answered their questions (98.8%), have understood the patients’ health issues (98.8%), have solved the patients’ health issues in a timely manner (98.8%), and had a respectful behavior towards them and their family members (98.8%) (Figure 3).

**Figure 4.**
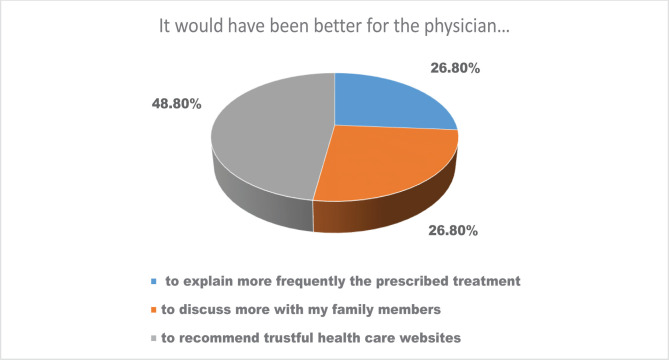
The distribution of the patients’ opinions regarding their expectations of the communication quality between physicians and patients.

In what concerns the future direction of the communication between physicians and patients, 26.8% of the patients would like their physicians to explain the prescribed treatment more and provide more information to their family members (26.8%). At the same time, 48.8% of the patients considered that their physicians should recommend trustful online information sources regarding their treatment and diagnosis (Figure 4).

### The results of objective 5

**Figure 5.**
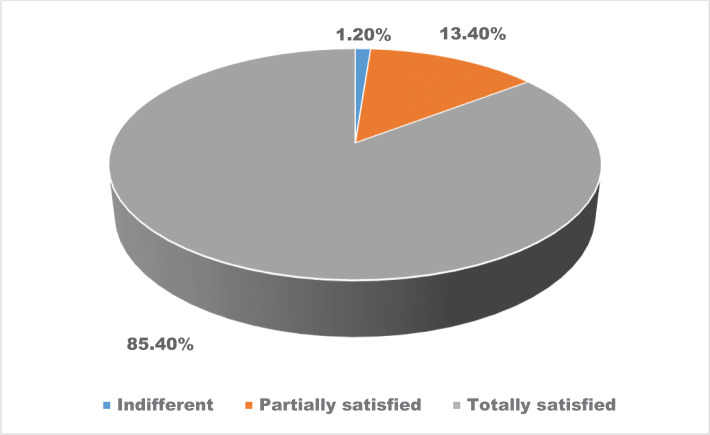
The distribution of the patients’ opinions regarding their general satisfaction of the communication between them and their physicians.

The general level of satisfaction of the patients regarding the communication between them and their physicians revealed that it should be based on the received quality of information, the established diagnosis, and the adequacy of the prescribed treatment. As such, the vast majority of patients have been satisfied (85.4%), 13.40% of the patients have been partially satisfied, and 1.20% of them adopted a careless behavior (Figure 5).

**Figure 6.**
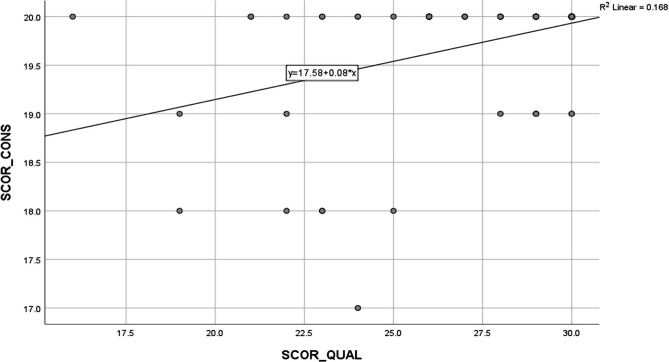
The correlation between the level of satisfaction of the consultation and the level of satisfaction of the physician-patient communication.

The correlation between the level of satisfaction regarding the consultation and the level of satisfaction regarding the communication between physicians and patients was positive and statistically significant, but at a moderate intensity (p=0.001, r=0.35). Therefore, a rise in the level of satisfaction related to the consultation will be proportional to the satisfaction level related to the communication between the patients and the physicians (Table 8 and Figure 6).

**Table 8.** The correlation between the level of satisfaction regarding the consultation and the level of satisfaction regarding the communication between physicians and patients.

**Correlation**	P *
**The consultation score (p<0.001) x the communication score (p<0.001)**	0.001, R=0.35

* – Spearman’s rho Correlation Coefficient, ** – Shapiro-Wilk Test

## Discussion

The aim of this study was to investigate the level of satisfaction related to the communication and the interaction of the patients with the physicians in an Infectious Diseases Hospital from Romania. The patients included in the study were aged between 18–28 years, were mostly females living in urban areas, and 41.5% had university degrees.

The main motivation mentioned by patients being hospitalized was the general bad general health. Before being admitted, most of them searched for information on the internet about a physician and similar symptoms. This defensive behavior is prevalent even more in the pandemic context when all the important activities occur online. Being afraid of getting infected with SARS-COV-2 is also a plausible motivation to search for online health care information and avoid interaction with any medical employee. As a matter of fact, since last year, the interaction with physicians has decreased but registered a rise in infectious disease services. Depending on the age category, the patients with the ages between 18–28 years and 29–39 years were more active in searching the internet for symptoms, in comparison with the patients who were in the age category of over 60. Obviously, the younger generations of patients are more IT literate and use the online environment when they get sick [6]. Nowadays, the internet has become a ubiquitous part of any person’s life that most people have access to and are comfortable using the internet for their information needs [7].

Further, this study’s findings also revealed that the satisfaction of the patients hospitalized in the Infectious Diseases Hospital mainly referred to the respectful behavior and the careful attention of the physicians. In fact, reaching a total score of 20 points out of 20, most patients considered that they had an excellent interaction with their physicians. Since there was a moderate and direct correlation between the interaction and communication variables (p<0.001, r=0.35), it may be concluded that satisfaction may be reflected in the communication factors as perceived by the physicians.

Most patients perceived their quality of communication with their physicians as suitable to the context, mentioning that physicians have answered their questions and solved the patients’ needs promptly. However, 26.8% of the patients would have liked their physicians to explain the prescribed treatment more and provide more information to their family members. Moreover, most patients also showed a high desire to engage in making the right decisions regarding their health and expressed their need for physicians to recommend trustful health websites (48.8%). As patients become more empowered [8], they will become more inclined to be involved in their health and decision-making [9]. However, the actions of the online information search of patients may have undesired consequences, for instance, patients being misinformed, become distressed, and increase the tendency of self-diagnosis and self-treatment [10].

The general level of satisfaction of the patients from a communication perspective was reflected in the received quality of information, the established diagnosis, and the adequacy of provided information of the prescribed treatment. As such, the vast majority of patients expressed high levels of perceived satisfaction. Investigating the extent to which patients are satisfied with health care services, and implicitly, with infectious diseases services, becomes clinically significant, as satisfied patients are more likely to comply with a prescribed treatment [11].

## Conclusions

Patient satisfaction is a complex concept, being influenced by many external factors. In practice, patient satisfaction is an indicator of health care quality. The outcomes of this study revealed that, in the context of infectious diseases services, patient satisfaction consists of two components: interaction and communication. As the interaction part proved to be highly appreciated, the communication component of satisfaction was partially appreciated. Patients mentioned that they would have liked to receive more information about the prescribed treatment and physicians to provide more information to their family members. It is also noteworthy to mention the need of the patients to become more empowered by using the recommended health websites by physicians.

## Acknowledgments

### Ethical approval

The approval for this study was obtained from the Ethics Committee of the Infectious Disease hospital, Baia Mare, Romania.

### Consent to participate

The participants were assured that the completed questionnaire would be anonymous and confidential and gave their consent for participating in this study.

### Conflict of interest

The authors declare that there is no conflict of interest.
